# *CRP* Genotypes Predict Increased Risk to Co-Present with Low Vitamin D and Elevated CRP in a Group of Healthy Black South African Women

**DOI:** 10.3390/ijerph15010111

**Published:** 2018-01-10

**Authors:** Pieter H. Myburgh, G. Wayne Towers, Iolanthé M. Kruger, Cornelie Nienaber-Rousseau

**Affiliations:** 1 Centre of Excellence for Nutrition, North-West University, 11 Hoffman Street, Potchefstroom 2520, North West Province, South Africa; cornelie.nienaber@nwu.ac.za; 2 Africa Unit for Transdisciplinary Health Research (AUTHeR), North-West University, 11 Hoffman Street, Potchefstroom 2520, North West Province, South Africa; wayne.towers@nwu.ac.za (G.W.T.); lanthe.kruger@nwu.ac.za (I.M.K.)

**Keywords:** 25-hydroxyvitamin D, 25(OH)D, calcidiol, calciferol, C-reactive protein, nutrigenetics, single nucleotide polymorphisms, SNPs, Tswana

## Abstract

Low 25-hydroxyvitamin D (25(OH)D) and elevated C-reactive protein (CRP) concentrations are independently associated with adverse health outcomes, including cardiovascular disease (CVD). Although an inverse association between these factors has been described, the underlying mechanisms remain unknown. We postulate that environment–gene interactions, through which 25(OH)D interacts with single nucleotide polymorphisms (SNPs) within the *CRP* gene, modulate CRP; that certain *CRP* genotypes predispose individuals to a co-phenotype of low 25(OH)D and elevated CRP concentrations; and that this co-phenotype is associated with higher CVD risk. Twelve *CRP* SNPs were genotyped, and both 25(OH)D and CRP were quantified, in 505 black South African women. Alarmingly, 66% and 60% of the women presented with deficient/insufficient 25(OH)D and elevated CRP concentrations, respectively. CRP concentrations were higher in individuals with lower 25(OH)D concentrations. However, no 25(OH)D–*CRP* genotype interactions were evident. Several genotypes were associated with an altered risk of presenting with the co-phenotype, indicating a genetic predisposition. Women presenting with this co-phenotype had higher blood pressure and increased anthropometric measures, which may predispose them to develop CVD. We recommend increasing vitamin D fortification and supplementation efforts to reduce inflammation among black women with vitamin D deficiency, thereby possibly curbing diseases contingent on the co-phenotype described here.

## 1. Introduction

Cardiovascular disease (CVD) is the leading cause of mortality, with 80% of the global burden carried by developing countries [1]. In South Africa, at the time of this study, one third of all deaths were attributable to CVD [2]. Since then, an increase in CVD-related deaths has been reported [3]. The role of chronic inflammation in the etiology of CVDs has recently come to the fore. Although the inflammatory response is regarded as crucial for survival, dysregulation of this process has detrimental effects, including the development of CVDs [4,5].

Several biochemical markers of inflammation have been identified, including tumor necrosis factor-α, interleukin 1 (IL-1) and 6 (IL-6), and C-reactive protein (CRP). CRP is by far the most widely investigated biomarker of low-grade systemic inflammation, as it has a stable half-life with well-established associations with disease, and has known cut-off values [6]. People with a basal CRP concentration greater than 3 mg/L are at higher risk of experiencing cardiovascular events [7]. Circulating CRP concentrations can be influenced by demographic factors (age, sex, and ethnicity) as well as environmental and behavioral factors (alcohol intake, diet, socio-economic status, and tobacco use) [8,9,10,11,12]. Among dietary factors, an inverse association between vitamin D and CRP has been established [13,14]. A recent meta-analysis [15] indicated that vitamin D supplementation decreased CRP concentrations by 1.08 mg/L (95% confidence interval (CI), −2.13, −0.03), while those with baseline CRP levels of >5 mg/L registered a more significant reduction of 2.21 mg/L (95% CI, −3.5, −0.92). Various genetic factors have been reported to affect CRP concentrations, with twin and family studies indicating substantial heritability of between 35% and 40% [16]. Furthermore, interactions have been observed between *CRP* single nucleotide polymorphisms (SNPs) and dietary intake influencing circulating CRP concentrations [17]. In contrast, infection with the human immune deficiency virus (HIV) is not associated with CRP [18], an observation re-established by the present study.

Black individuals tend to have higher circulating CRP concentrations than those from other groups [19]. African Americans, included in a meta-analysis, presented with CRP concentrations of 2.6 mg/L compared to 2.51 mg/L in Hispanics, 2.03 mg/L in white Americans, and 1.01 mg/L in East Asians [20]. Elevated CRP concentrations are also typical in black South Africans [17]; comparisons show them to be higher (3.61 mg/L) than those of their white (1.13 mg/L) compatriots [21].

Ethnicity also affects vitamin D status. Darker skin (type V and VI skin on the Fitzpatrick scale [22]), as observed in African individuals, seems to have originated in persons living in areas with high ultra-violet (UV) radiation (UVR) [23]. Even though it is a photoprotective mechanism, darker skin reduces the synthesis of vitamin D [24] as measured by circulating 25-hydroxyvitamin D (5(OH)D), or calcidiol, concentrations. Darker-skinned humans require approximately six times more UVR exposure than their fairer-skinned counterparts to produce similar amounts of vitamin D [25]. South Africa, ranging between the ~22° and 34° southern latitudes, experiences relatively intense UVR, although not as extreme as at the equator [25]. Black South Africans present with lower 25(OH)D concentrations than dark-skinned people living closer to the equator [26]. The rapid urbanization observed in most African countries, resulting in reduced exposure to UVR, has been proposed as a major contributing factor to the low 25(OH)D status observed in black Africans [27]. Other risk factors associated with low 25(OH)D status include age, obesity, HIV infection, and smoking [28,29,30].

Apart from influencing inflammation, vitamin D itself is related to disease risk. 25(OH)D has preventive effects on a range of chronic maladies, including CVD [31]. Two meta-analyses found an increase in the risk of developing ischemic heart disease, as well as an augmented risk of symptomatic ischemic stroke for the participants in the lowest quartiles of 25(OH)D concentrations [32,33]. Another meta-analysis reported an inverse association between 25(OH)D concentrations and the risks associated with all-cause mortality (relative risk (RR) of 1.35 (1.22–1.49)) and CVD (RR: 1.35 (1.13–1.61)) [34].

As elevated concentrations of CRP are associated with increased CVD risk, whereas CVD risk is reduced with elevated levels of 25(OH)D, a hypothesis has been proposed that 25(OH)D might influence CRP [35]. However, there is no evidence of a direct pathway by which 25(OH)D or its metabolized product, 1,25-dihydroxyvitamin D (or calcitriol), affects the expression of *CRP* (or vice versa) [13]. Vitamin D-mediated mechanisms for a reduction in vascular damage have been proven experimentally, with the inhibition of cholesterol uptake by macrophages and the suppression of the renin gene [14]. In vitro studies have also described the diminished production of IL-6 in monocytes treated with vitamin D_3_ (cholecalciferol, or calciol) compared to untreated cells [35]. IL-6, synthesized by macrophages, is transported to the liver, where the transcriptional activation of *CRP* is mediated via Signal Transducer and Activator of Transcription factor 3 (STAT3) [36,37]. These physiological mechanisms might, therefore, act as potential indirect pathways by which vitamin D and its metabolites could influence CRP concentrations. Thus, having elevated CRP as well as low 25(OH)D concentrations, which are both independently associated with increased CVD risk, may exacerbate disease development.

Because black individuals tend to be predisposed to lower 25(OH)D and higher CRP concentrations, investigations that involve this particular population will increase the chance of observing sufficient numbers of the phenotypes of interest. An inverse association between 25(OH)D and inflammation has been described, but the underlying mechanisms remain unknown [13]. Moreover, it has to be established whether vitamin D status influences *CRP* differently in individuals harboring specific *CRP* genotypes in modulating CRP concentrations. We determined whether vitamin D status possibly interacts with these *CRP* genotypes to affect the CRP concentrations. In addition, we tested whether *CRP* SNPs affected 25(OH)D concentrations. Studying environment–gene interactions is important, as identifying these interactions and modifying behaviors accordingly could improve health outcomes. Furthermore, we determined whether specific *CRP* genotypes predispose individuals to a co-phenotype of low 25(OH)D and elevated CRP, as well as whether this co-phenotype is associated with a higher CVD risk in black South African women. This research is important, because unraveling possible mechanisms for the observed relationship between vitamin D and CRP leads to a better understanding of the foundation of this relationship and paves the way for designing targeted approaches to treat the corresponding elevated CRP concentrations and low 25(OH)D concentrations in black individuals. This research might also indicate whether efforts to increase responsible sunlight exposure and include more vitamin-D-rich foods in their diet are, and/or whether supplementation with vitamin D is, desirable for black South African women.

## 2. Materials and Methods

### 2.1. Ethical Considerations

For this cross-sectional investigation, we used data collected for the South African arm of the Prospective Urban and Rural Epidemiology (PURE-SA) study, at baseline (2005). Ethical approval, in accordance with the Declaration of Helsinki as revised in 2004 [38], was obtained for the larger study from the Health Research Ethics Committee of the Faculty of Health Sciences, North-West University (NWU–HREC, ethics number: 04M10). Ethical approval was also granted for this affiliated study (ethics number: NWU-00004-17-A1). Goodwill permission was granted to the PURE study by mayors, household heads, community leaders of the communities included, and tribal chiefs before the research started. Participants were well-advised about the research project and were asked to sign an informed consent form, after sufficient time for reflection, to indicate their agreement to take part in the study. Subjects could withdraw at any time, or withhold any information they were not comfortable sharing.

### 2.2. Research Design and Study Population

The PURE-SA study aims to investigate the development of chronic lifestyle diseases, with a focus on CVDs, by stratifying populations at different levels of urbanization [39]. Four communities were selected in 2005 in South Africa, based on their degree of urbanization, and grouped into either being urban (Location A) or rural (Location B). The initial sampling strategy is explained elsewhere [40].

Eligible participants were all apparently healthy adults who were older than 30 years. On the day of enrollment, individuals with elevated body temperatures (above 38 °C) were excluded to reduce the number of volunteers with acute infections. Further exclusion criteria were that potential volunteers were not allowed to use chronic medication, to have any known lifestyle disease, be pregnant or lactating, or to have a known infection, such as tuberculosis-causing agents and/or the human immune deficiency virus (HIV) (details in [40]). Sampling was conducted between August and November 2005, which is late winter to late spring in the southern hemisphere. Of 6000 individuals screened, 2010 were included at baseline. The 25(OH)D status of a subset of 660 randomly selected women was determined, because of constrained budgets and the fact that women are more likely to develop skeletal disorders associated with low 25(OH)D status.

### 2.3. Biochemical and Blood Pressure Measurements

Fasting participants, defined as *sans* food and beverages (water permitted) from the evening before enrollment, arrived at the study site, upon which professional nurses obtained blood samples. Blood tubes were centrifuged at 2000× *g* for 15 min at 10 °C. Plasma, serum, and buffy-coat were aliquoted and snap-frozen on dry-ice pellets before storage at −70 °C. Serum high-sensitivity CRP concentrations were measured on a Sequential Multiple Analyzer Computer using a particle-enhanced immunoturbidometric assay (Konelab TM auto analyzer, Thermo Fisher Scientific, Vantaa, Finland). Total 25(OH)D (sum of D_2_ and D_3_) in serum was quantified using a Roche Elecsys 2010 COBAS system (functional sensitivity: 10.0 nmol/L; Roche Diagnostics, Indianapolis, IN, USA). Lipograms, including high-density lipoprotein cholesterol, triglycerides, and total cholesterol, were performed using a Konelab 20i auto analyzer (Thermo Fisher Scientific, Vantaa, Finland). The Friedewald equation was used to calculate the low-density lipoprotein cholesterol (LDL-c) in those with triglyceride concentrations below 400 mg/dL [41]. Research nurses—trained in voluntary counseling and the testing of HIV, adhering to the UNAIDS/WHO policy statement on HIV testing as well as the protocols set by the National Department of Health of South Africa—gave all participants pre-test counseling. Volunteers could then decide whether they wanted to be tested, with specific signed informed consent obtained for HIV testing after pre-test counseling. HIV determination was conducted using a rapid First Response HIV 1-2.O card test (Transnational Technologies Inc., PMC Medical, Nani Daman, India). Persons testing positive were re-tested using a second card test, developed by Pareeshak (BHAT Bio-Tech, Bangalore, India) to affirm HIV status. All participants, irrespective of their HIV status, were given post-test counseling individually. Blood pressure was measured in duplicate with an Omron automatic digital blood pressure monitor (Omron HEM-757) after 5 min of sitting in a calm environment.

### 2.4. Anthropometric Measurements

Body weights (kg) were measured twice on calibrated and tared scales, with the mean recorded, while participants were lightly clothed and their arms hanging freely at their sides. Heights (cm), with volunteers’ heads in the Frankfort plane, bodies fully extended while inhaling, were measured twice to the nearest 10 mm, using stadiometers, and the mean was reported in meters. Body mass index was computed as kg/m^2^.

### 2.5. Factors Pertaining to Lifestyle

Participants responded to various interviewer-administered questionnaires in a language of their choice. These test instruments included questions on medical history and tobacco use. Nutritional information from the previous 30 days was obtained using validated, interviewer-based quantitative food frequency questionnaires (qFFQs) and employing food portion books standardized for the population under investigation [42]. qFFQs’ data were entered into FoodFinder 3 (Medical Research Council, Tygerberg, South Africa) and analyzed by the Medical Research Council of South Africa for nutrient content.

### 2.6. Genetic Analyses

Determination of the genotypes via a BeadXpress analysis was performed by the National Health Laboratory Service located at the University of the Witwatersrand, Johannesburg. For details on the genetic analyses, please refer to Nienaber-Rousseau et al. [17].

### 2.7. Environmental Data

Locations A and B were compared using the means of data from 1 August (late winter) to 1 December 2005 (late spring). Environmental factors were investigated using satellite data obtained from an online repository, Giovanni [43]. Average mean temperature, UV index, erythemal dose rate, and total ozone column were downloaded as Google Earth data files (.kmz files).

### 2.8. Statistical Analyses

As previously mentioned, 25(OH)D concentrations were available for 660 randomly selected women. Only individuals for whom 25(OH)D concentrations, CRP concentrations, *CRP* genetic data, and all anthropometric markers were available were included in our statistical analyses (*n* = 534). Furthermore, women with 25(OH)D or natural log-transformed (ln)CRP concentrations greater than 5 standard deviations were excluded as outliers. The final number of participants was 505. Statistical analyses were conducted in R [44].

Numeric variables were visually inspected for normality as well as measures of skewness. Non-parametric variables (CRP) were log-transformed, yet still reported as median and interquartile ranges. Women were grouped as two phenotypes: the case phenotype including individuals with deficient/insufficient 25(OH)D (<75 nmol/L) and elevated CRP (>3 mg/L), and a control phenotype consisting of the remaining volunteers. Pairwise comparisons using the Wilcoxon ranked-sum test were performed to identify significant differences in stratified continuous variables. Comparative tables were created with the compare Groups library in R [45] using non-parametric methods. Spearman correlations were used for testing associations between numeric variables. Multivariate linear models predicting lnCRP concentrations from continuous 25(OH)D values were constructed using backward step-wise linear regressions and evaluated based on the Akaike Information Criterion (AIC). Adherence to Hardy–Weinberg equilibrium (HWE) was tested by a chi-squared (χ^2^) test using SNPassoc, and linkage disequilibrium (LD) was calculated using the LDheatmap library of the R package.

Variables identified in regression analyses were evaluated for co-linearity. Possible environment–SNPs interaction was determined using SNPassoc [46] while including covariates obtained from the linear regression model. To determine whether *CRP* SNPs influence vitamin D status, 25(OH)D was used as the dependent variable. To evaluate the risk associated with certain *CRP* SNPs to present with the phenotype of low 25(OH)D combined with elevated CRP concentrations, the case and control phenotypes were entered as dependent variables.

Where applicable, *p*-values were adjusted using the methods suggested by Bonferroni. Significance was defined as an α level of 0.05.

## 3. Results

### 3.1. Association of 25(OH)D Concentrations/Status with Circulating CRP Concentrations

**Figure 1:**
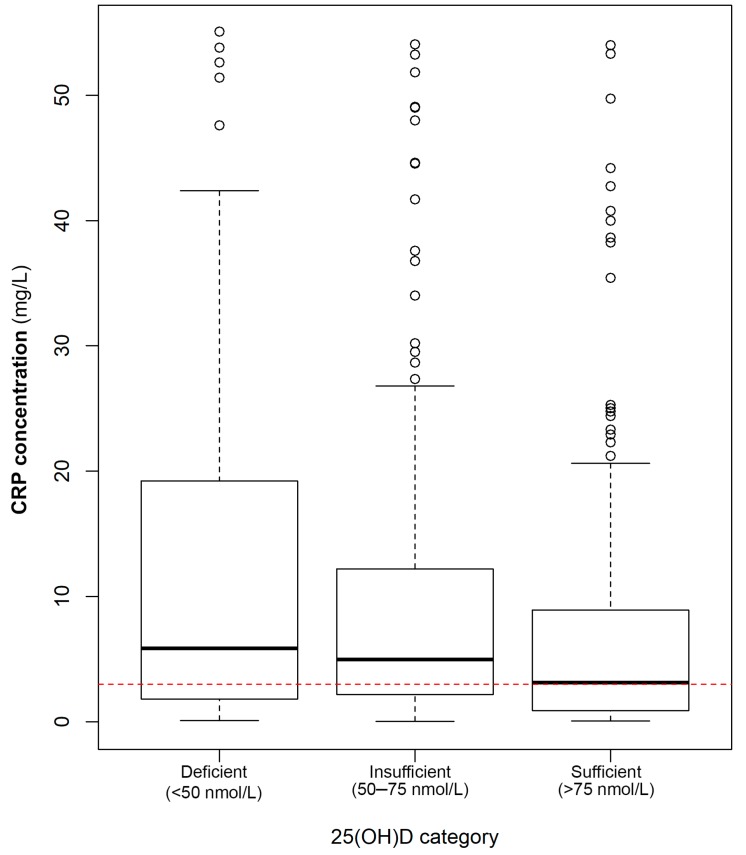
Median C-reactive protein (CRP) concentrations across different categories of 25(OH)D status (*p* = 0.001). Pairwise Wilcoxon ranked-sum test with Bonferroni adjustment revealed that women with sufficient 25(OH)D concentrations had significantly lower CRP concentrations than those with deficient or insufficient 25(OH)D. Outliers depicted as open circles. The red dashed line indicates the cut-off value for CRP concentrations with elevated CRP being greater than 3 mg/L.

Median concentrations for 25(OH)D and CRP were 68.2 nmol/L and 4.13 mg/L, respectively, indicating that the 25(OH)D status of the women in our cohort was insufficient, while they also presented with elevated inflammation based on CRP. CRP concentrations decreased across increasing 25(OH)D categories, with the median CRP concentration being significantly lower in the sufficient 25(OH)D group compared to both the deficient and insufficient subdivisions (Figure 1). The largest variability in CRP concentrations was observed for those in the 25(OH)D-deficient category, with decreasing variability in the insufficient and sufficient groups. In the population investigated, 42% (*n* = 216) of individuals presented with both 25(OH)D concentrations lower than 75 nmol/L and elevated CRP concentrations above 3 mg/L (case phenotype).

In Table 1, we have summarized the demographic characteristics of the case and control phenotypes: 25(OH)D concentrations decreased with age. The distribution between rural and urban cases and controls was similar. In addition, the environmental exposure that could have influenced vitamin D status did not differ between the rural or urban areas. Individuals representing the case phenotype were significantly older. Similar distributions were also observed in respect of smoking and HIV status. The median dietary intake of vitamin D sources did not differ for the two groups either.

Differences in CVD risk markers between the two phenotypes are presented in Table 2. Those representing the control phenotype had lower blood pressure. However, heart rate was similar in the two groups. Anthropometric markers indicated that cases had significantly higher body weight and increased waist circumference. More obese individuals were also observed in the case phenotype. HDL-c was significantly higher in the control group; however, after adjusting for age, waist circumference, and LDL-c, the significance was eliminated. Furthermore, no statistical differences were observed for LDL-c, triglycerides, or energy intake, although energy intake was lower for the controls than for the case phenotypes.

### 3.2. Quantification of the Associations of 25(OH)D with CRP Concentrations

Spearman correlation analyses (results not shown) revealed that 25(OH)D was inversely, albeit weakly (ρ > −0.20; *p* ≤ 0.05), associated with age. No other factors were associated with 25(OH)D with a correlation greater than 0.20, so that these are not reported here except for the correlation with CRP presented later. CRP was moderately associated with anthropometric markers (all ρ > 0.30; *p* < 0.05) and lipid profile markers of which LDL-c (ρ = 0.13; *p* < 0.05) presented with the strongest correlation. Similar to vitamin D status, other variables did not correlate strongly with CRP even though these correlations were statistically significant.

A weak, yet significant, negative correlation was observed between 25(OH)D and CRP (ρ = −0.15; *p* < 0.05). Converting 25(OH)D from nmol/L to mg/L, using a conversion factor of 0.0004 (Equation (1)), indicated that a one-unit increase in 25(OH)D was associated with a 0.15 mg/L decrease in CRP concentration. Vitamin D intake did not correlate with 25(OH)D or CRP concentrations.

(1) $$\begin{array}{c} 1\;\mathrm{nmol}/L\;25\left(\mathrm{OH}\right)D=0.4\;\mathrm{ng}/\mathrm{mL}\;25\left(\mathrm{OH}\right)D=0.0004\;\mathrm{mg}/L\;25\left(\mathrm{OH}\right)D\\ 0.0004\;\mathrm{mg}/L\;25\left(\mathrm{OH}\right)D\equiv -0.15\;\left(\mathrm{CRP}\right)\mathrm{mgL} \end{array}$$

The linear relationship between 25(OH)D as a factor influencing lnCRP concentrations was modelled in two ways: for the first model, we adjusted for age; and in the second, we adjusted for age, anthropometrical marker (see discussion below), and LDL-c. These covariates were chosen owing to the likelihood of these variables influencing the model based on their previous association with CRP concentrations, as well as having the lowest AIC score. As there is a large degree of co-linearity between anthropometric markers, each marker (i.e., BMI, waist and hip circumference, and weight) was entered into the model separately, and models were evaluated based on their resulting AIC value. The lowest AIC value was observed for waist circumference and LDL-c; therefore, these markers were used as a proxy for all other anthropometric and lipid profile markers, respectively. Dietary sources of vitamin D did not affect the model (*p* > 0.05). The unadjusted model (Model 1) accounted for 2.1% of the variance of lnCRP (calculated from adjusted R^2^ values; *p* = 0.001). For Model 1, a 1.1% reduction in CRP concentration (converting lnCRP to CRP by using (e^β^ − 1) × 100)) for each 1 nmol/L increase in 25(OH)D was observed. In Model 2, when adjusting for age, waist circumference, and LDL-c, 1.8% of the lnCRP variation could be explained (*p* < 0.00001). The inverse relationship between vitamin D status and CRP was slightly intensified when controlling for the covariates. Here, for each 1 nmol/L increase in 25(OH)D, CRP decreased by 1.1%. Excluding individuals with CRP concentrations above 10 mg/L—the clinical cut-off point for acute inflammation—resulted in similar trends being observed (0.71% and 0.82% reduction per 1 nmol/L increase of 25(OH)D for unadjusted and adjusted models, respectively; results not shown).

### 3.3. SNP Interaction

**Figure 2:**
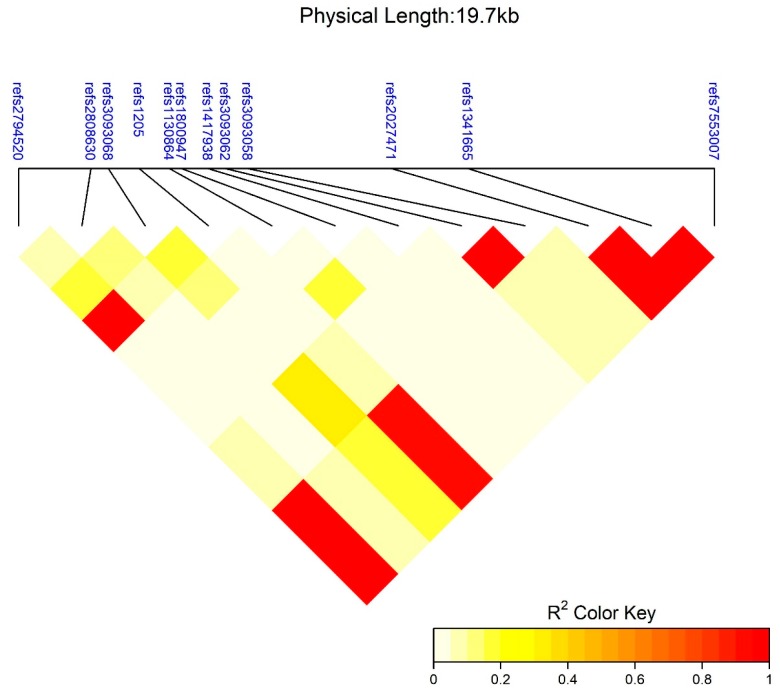
Linkage disequilibrium heatmap indicating linkage between 12 single nucleotide polymorphisms (SNPs) on the *CRP* gene.

All genotyped SNP frequencies reflected the assumptions of what would be expected under Hardy–Weinberg equilibrium. Previously, in our population, LD was reported between rs2027471 with rs1341665 and rs3093058 with rs3093062 [17]. In the subset of women studied here, the same LD pattern (Figure 2) was observed. Here, linkage was also detected for a haplogroup linking rs7553007, rs1341665, rs2027471, rs1205, and rs2794520 (Figure 2).

Using the association analyses provided by the SNPassoc package in R, trends were investigated based on models of interaction between 25(OH)D concentrations with *CRP* SNPs on lnCRP concentrations. Associations were investigated under co-dominant, dominant, additive, and recessive genetic models, and the genetic model with the lowest AIC values was included (Supplementary Table S1). The minor alleles of the five SNPs (i.e., rs7553007, rs1341665, rs2027471, rs1205, and rs2794520) in high linkage were previously reported to be associated with decreases in circulating CRP [17], which was echoed by our results. Three SNPs were also significantly associated with increased CRP concentrations: rs3093058, rs3093062, and rs3093068.

To quantify whether 25(OH)D concentrations interacted with any of the 12 *CRP* SNPs to affect lnCRP concentrations, factorial analyses of co-variance with age, LDL-c, and waist circumference as covariates were performed. In the 12 SNPs investigated, 96.0% of the genetic variation could be grouped into six haplotypes. No interactions were observed between 25(OH)D concentrations and the identified haplotypes to affect lnCRP concentrations (*p* for trend = 0.68). No significant associations between *CRP* SNPs and 25(OH)D were found under either the co-dominant or dominant model.

To investigate whether a genotype was associated with an increased risk of presenting with either deficient or insufficient 25(OH)D and elevated CRP concentrations, odds ratios (OR) were calculated using the SNPassoc library in R for each of the 12 SNPs while adjusting for age, LDL-c, and waist circumference (Table 3). The minor alleles of SNPs previously associated with significant increases in CRP concentrations were found to be at higher odds of co-presenting with insufficient/deficient 25(OH)D concentrations and vice versa. The minor alleles of the five SNPs in LD were associated with a reduced risk of presenting with the phenotype of inadequate 25(OH)D combined with elevated CRP concentrations (cases) compared to the phenotype presenting with either sufficient 25(OH)D or normal (<3 mg/L) CRP concentrations. Of these, rs3093068, rs3093062, and rs3093058 presented with increased odds (1.54, 1.64, and 1.67, respectively), while reduced odds were observed in rs2794520 and rs7553007 (0.65 and 0.67, respectively) for individuals harboring the minor alleles. A trend towards significance for ORs for carriage of the minor alleles at two *CRP* SNPs (rs2027471 and rs1341665) to present with the co-phenotype was observed (OR: 0.05; *p* > 0.05).

## 4. Discussion

In this research, we confirmed the existence of an inverse relationship between CRP and vitamin D and attempted to unravel the mechanisms involved. From this study, we know that vitamin D status does not directly modulate CRP via *CRP* SNPs nor do *CRP* SNPs influence vitamin D, even though these SNPs influence CRP concentrations (data presented elsewhere). Since we found that the double phenotype of high CRP and low vitamin D was associated with particular *CRP* SNPs, we hypothesized that negative feedback mechanisms were at play (will be described later). In cases where these genotypes were co-observed with low 25(OH)D concentrations, poorer CVD markers were also observed, such as elevated blood pressure.

The majority (65.5%) of the South African women in our cohort had 25(OH)D concentrations lower than the recommended 75 nmol/L. Moreover, 42.8% of them presented with the co-burden of low vitamin D status combined with elevated CRP concentrations. Having deficient or insufficient 25(OH)D and elevated CRP concentrations was previously shown to increase the risk of developing various chronic non-communicable conditions, such as CVD [5,31,34]. In our study, volunteers classified as cases (those presenting with both low vitamin D status and high CRP concentrations) had significantly higher blood pressure and anthropometrical markers (even when adjusting for waist circumference), precursors in the etiology of CVD, than controls. We established that vitamin D correlated inversely with CRP, which is in accordance with a previous report [13]. Moreover, we found that CRP decreased within increasing strata of 25(OH)D categories as recommended by the Endocrine Society [47] and confirmed this inverse association with our regression models. Furthermore, we proposed that 25(OH)D might interact with SNPs located on the *CRP* gene, thereby influencing CRP concentrations. Contrary to our hypothesis, none of the *CRP* SNPs investigated showed any interactions with circulating 25(OH)D; thus, vitamin D does not modulate *CRP* genotypes to influence CRP concentrations. The inverse association between vitamin D and CRP is therefore not due to nutrigenetic effects. A recent Mendelian association study also found a lack of association between 25(OH)D and genetic markers influencing CRP concentrations [13] even though they detected a negative correlation between 25(OH)D and CRP concentrations.

We also investigated whether *CRP* SNPs influenced 25(OH)D concentrations directly to learn whether a possible unknown backward feedback mechanism might exist or whether certain *CRP* SNPs predispose individuals to having heightened inflammation, with concurring maladies, leading to reduced UV exposure resulting in altered vitamin D status, but found no associations. We then determined whether harboring CRP genotypes could increase the odds of co-presenting with both high CRP and low vitamin D status. Carriage of the minor allele at three SNPs (rs3093068, rs3093058, and rs3093062) was associated with increased odds, while harboring the variant allele at two SNPs (rs2794520 and rs7553007) resulted in lower odds to present with the phenotype of insufficient/deficient 25(OH)D (<75 nmol/L) and elevated CRP concentrations (>3 mg/L). This is, to our knowledge, a novel addition to the existing literature, as these genetic effectors were not previously reported in this context.

How blood pressure is influenced by vitamin D status remains inconclusive [48], although there are suggestions of causative pathways [49]. Li [50] hypothesized that vitamin D could have an influence on the renin–angiotensin system (RAS), which was substantiated by a study that reported how low concentrations of 25(OH)D upregulated the RAS [51]. In our population, 25(OH)D was reported to be associated with carotid wall thickening and arterial stiffness [52], both being attributes observed in individuals with increased CRP concentrations. This is a possible mechanism whereby low 25(OH)D could result in increased arterial stiffness, and in turn result in increased blood pressure [53], leading to elevated CRP concentration by negative feedback mechanisms. Individuals with the case phenotype were also investigated in another population, where cases had worse pro-inflammatory marker panels than controls [54]. However, with increasing CRP concentrations, it was reported that the anti-inflammatory effects of vitamin D decreased substantially and most of the other pro-inflammatory markers were upregulated [54]. These risk factors are further exacerbated by the presence of abdominal adiposity observed in our population [55], thereby further increasing the risk of developing CVD by means of increased pro-inflammatory factors (such as IL-6) released by adipose tissue. Another possibility is that low vitamin D status has harmful side effects that are pro-inflammatory themselves; alternatively, synergistic effects between these two factors may exist, explaining the co-existence of low vitamin D with high CRP, as was the case in Kuwaiti women [54]. Low concentrations of 25(OH)D have been linked to an increased risk of developing CVD [56], although definite conclusions remain ambiguous [57]. Similarly, CRP has been strongly associated with increased CVD risk [6]. Future studies should explore possible mechanisms for the inverse association between 25(OH)D and CRP further, as well as whether the presence of both low vitamin D status and inflammation might heighten disease risk.

Differences in 25(OH)D were observed among individuals residing in the two different locations (A and B) that were investigated here (Supplementary Table S2). Although 25(OH)D and CRP values were closer to the recommended concentrations in rural participants, neither median 25(OH)D nor CRP concentrations met the recommended guidelines in both population subdivisions. Rural-urban differences in 25(OH)D disappeared when adjustments for age were made. Although age was reported as a non-significant contributor to our linear models, it was included in analyses as a possible covariate based on four previously reported reasons. First, aging results in decreased concentrations of 7-dehydrocholesterol in the epidermis, which in turn reduces the response to UV light and subsequently results in decreased formation of pre-vitamin D_3_ [58,59]. Second, a decline in absorption, transport, or liver hydroxylation of orally ingested vitamin D sources was reported in older individuals [60]. Increased frailty with advancing age may also result in individuals spending less time outdoors, affecting their exposure to UV sources. Lastly, age, sex, and ethnicity are recommended factors to adjust for when conducting predictive analyses for CRP [12], with sex and ethnicity controlled for in our black, female population. It could, however, be argued that environmental and climate differences, such as reduced UVR, could have affected 25(OH)D concentrations between individuals located in the two different locations of our study. These places, A and B, whence we drew our samples, were on similar latitudes; when measured using the equator as a reference the difference between them was 14 km. In terms of elevation above sea level, they differed by less than 80 m. The likely differences in UV radiation were therefore small. Data pertaining to environmental factors influencing vitamin D synthesis in individuals were not available from ground observations in these localities and, therefore, satellite observations were used. Similar mean average temperatures near the earth’s surface, UV indices, erythemal dose rates, and total ozone columns were observed between the two areas from which we recruited volunteers. Another factor that could have affected 25(OH)D concentrations in rural participants is differences in lifestyle, as observed in Asian [61], other African [62], and European [63] populations. As no data were available pertaining to sun exposure in our study, no inference about differences in lifestyle was possible. Low 25(OH)D concentrations have also been linked to an increased risk of obesity [31]; however, for the population investigated similar anthropometric markers were observed across differing 25(OH)D status. The lack of association between BMI and 25(OH)D concentrations was reported in another South African study, where recruitment was done in a province that neighbors the one from which we selected our volunteers [64]. This may indicate that the correlation between anthropometric markers and 25(OH)D status does not apply to black individuals living in South Africa, which necessitates further investigation.

Nutritional intake of vitamin D in our population did not differ between the three 25(OH)D categories and was well below the recommended 15 µg/day [27]. Dietary vitamin D also failed to influence the linear regression modeling of lnCRP concentrations, which might be due to the extremely low dietary intake observed. A low intake of dietary vitamin D is common in African populations, with only margarine being fortified with vitamin D in South Africa [27]. Dietary sources of vitamin D did not significantly contribute to vitamin D status [65], as ingested vitamin D is more readily excreted [66]. This result aligns with the fact that the primary factor contributing to 25(OH)D concentration is exposure to UV light [24], resulting in vitamin D_3_ (cholecalciferol or calcidiol) synthesis from 7-dehydrocholesterol (a precursor of cholesterol); after hydroxylation by the liver and kidneys, vitamin D_3_ becomes 25(OH)D and then 1,25-dihydroxyvitamin D. That said, a study controlling caloric intake of obese women showed that replenishment of 25(OH)D by supplementation with vitamin D at 2000 IU per day led to participants in that group losing more weight, having smaller waist circumferences, and a 46% higher reduction in CRP concentrations compared to those in a placebo group [67]. Because a placebo group was included in this other study, the reduction in CRP was attributed to 25(OH)D values stabilizing at sufficient levels [67], not simply a reduction of body composition markers. We attributed the elevated CRP concentrations observed in our urban population to increased abdominal adiposity, as urban individuals had increased waist circumferences. In their review, Brooks et al. [68] reported that the association between inflammation, as measured by CRP, and abdominal adiposity is highly correlated, even when correcting for BMI. Women with increased waist circumferences were previously reported to be at greater risk of co-presenting with elevated CRP concentrations [69], with our results indicating that waist circumference was also the largest effector contributing to CRP concentrations. Reductions in abdominal adiposity in response to dietary interventions were previously reported to reduce CRP concentrations [70]. These dietary interventions included supplementation with fish oil tablets, and although not stated in the original work, fish oil naturally contains bioavailable vitamin D, which could have contributed to the reduction in CRP concentrations [71]. Here, we report a predicted 0.15 mg/L reduction in CRP concentration with a 1 nmol/L increase in 25(OH)D concentrations as determined from Spearman correlation. Recommendations intended to achieve sufficient 25(OH)D status should, therefore, aim to include responsible guidelines for exposure to sunlight, as well as an increase in the intake of good dietary sources of vitamin D to improve health in a country such as South Africa, which has a history of health policies failing its citizens.

At the time of our study, South Africa was rife with HIV denialism by government and stigmatization of seropositive individuals, which resulted in a large segment of the population not knowing their HIV status. Overall, 8.1% of our study population was first diagnosed as being HIV-positive during our investigation. Median values, when excluding HIV-positive individuals, were similar, in terms of both 25(OH)D and CRP concentrations, to those of the whole group. Both 25(OH)D [72] and CRP [73] concentrations can be affected by the use of anti-retroviral (ARV) treatments; however, as these individuals were first diagnosed during this study, they were not receiving ARVs and were therefore not excluded.

Our study was not without limitations. Because gender is a factor that contributes to both 25(OH)D and CRP concentrations, including men in our sample would have made the study more informative. Future studies should aim to explore the relationship between vitamin D and inflammation in men as well as women. Furthermore, no data were available on sun exposure time, which could have contributed to explaining the variance in 25(OH)D concentrations. However, measuring 25(OH)D concentrations is a strength of our study as it avoids the necessity for UVR, sun exposure time, and even dietary vitamin D intake data. The cohort was also randomly selected, without prior genetic screening. Future studies, including more extensive numbers of minor allele carriers, could establish associations between SNPs where our population had too few genotypes, resulting in lowered statistical power. Because 1,25(OH)2D is epigenetically active [74], future investigations to explore a possible mechanism to explain the anti-inflammatory effects of vitamin D could also incorporate epigenetics.

Our study cohort was very well-described, with data available on daily vitamin D intake, and was particularly well-characterized in terms of demographic, genetic, and biochemical factors that could address the variance in circulating CRP concentrations. The unique population investigated here was ideal, as individuals presented with both low 25(OH)D and elevated CRP concentrations. Including both CRP concentrations, known to fluctuate quite extensively, and genetic constants with known effects on CRP phenotype further strengthens the data presented here.

## 5. Conclusions

Disturbingly, 43% of our female cohort presented with both elevated CRP concentrations and deficient/insufficient 25(OH)D levels; because the combination is linked to CVD, this may compound their disease risk and predispose them to future disease. A negative association was identified between 25(OH)D and circulating CRP concentrations, but no vitamin D–gene interactions were observed between common SNPs on the *CRP* gene and 25(OH)D in this study. Moreover, *CRP* SNPs did not influence vitamin D status. Several genotypes were, however, associated with an altered risk of presenting with the co-phenotype of insufficient/deficient 25(OH)D and elevated CRP, indicating that a genetic predisposition exists. The present research extends past work by demonstrating that the link between 25(OH)D and CRP is not associated with vitamin D–*CRP* gene interactions and that other pathways need to be investigated. This finding is important, as it offers a starting point for unraveling the possible mechanisms for the previously reported inverse relationship between 25(OH)D and CRP, which in turn may ultimately result in therapeutic and policy recommendations to combat CVD.

This paper highlights the necessity of public health efforts in South Africa to assist women to achieve sufficient vitamin D status through responsible exposure to sunlight, increased intake of natural and fortified dietary sources of vitamin D, and for those who require it, vitamin D supplementation. Improved vitamin D status would reduce inflammation and thus possibly curb diseases contingent on both low vitamin D and elevated CRP: the co-phenotype described here.

## Figures and Tables

**Table 1:** Comparisons of demographical and biochemical factors in the cohort stratified by control and case phenotypes.

Variable	Controls (*n* = 289, 57.2%)	Cases (*n* = 216, 42.8%)	*p*-Value
Urban/Rural	144 (49.8%)/145 (50.2%)	126 (58.3%)/90 (41.7%)	*NS*
Age (years)	53.0 (49.0; 59.0)	56.0 (51.0; 63.0)	<0.001
Smoking status: Former/Current/Abstainer	6 (2.10%)/138 (48.3%)/142 (49.7%)	7 (3.24%)/96 (44.4%)/113 (52.3%)	*NS*
HIV positive/negative	26 (9.03%)/262 (91.0%)	15 (6.98%)/200 (93.0%)	*NS*
Vitamin D intake (µg/day)	2.00 (1.02; 3.30)	2.05 (1.02; 3.66)	*NS*
Menorhea/Amenorhea	64 (23.0%)/214 (77.0%)	37 (17.2%)/178 (82.8%)	*NS*

Data presented as median (25th and 75th percentiles) for continuous data and number of observations (percentage) for categorical data. Abbreviations: 25(OH)D, 25-hydroxyvitamin D; HIV, human immune deficiency virus; *NS*, not significant (*p* > 0.05).

**Table 2:** Markers of cardiovascular disease (CVD) risk among the control and case phenotypes.

Variable		Controls (*n* = 289, 57.2%)	Cases (*n* = 216, 42.8%)	*p*-Value	Adjusted *p*-Value
Systolic blood pressure (mmHg)		133 (118; 148)	138 (124; 159)	<0.001	<0.01
Diastolic blood pressure (mmHg)		87.0 (78.0; 96.0)	91.0 (83.8; 101)	<0.001	<0.01
Heart rate (BPM)		73.0 (65.0; 83.0)	72.0 (63.0; 85.0)	*NS*	*NS*
Waist circumference (cm)		79.3 (70.8; 87.7)	86.4 (74.8; 95.5)	<0.001	<0.001
Hip circumference (cm)		98.0 (89.5; 106)	106 (94.1; 119)	<0.001	0.01
WHR		0.81 (0.76–0.87)	0.81 (0.76–0.86)	*NS*	*NS*
Weight (kg)		60.2 (52.1; 72.8)	72.0 (56.5; 85.5)	<0.001	<0.01
BMI (kg/m^2^)		24.7 (21.3; 29.0)	29.9 (23.3; 35.2)	<0.001	<0.001
BMI category	*Underweight*	29 (10.0%)	22 (10.2%)	<0.001	<0.001
	*Healthy*	122 (42.2%)	45 (20.8%)		
	*Overweight*	80 (27.7%)	42 (19.4%)		
	*Obese*	58 (20.1%)	107 (49.5%)		
Total cholesterol (mmol/L)		5.21 (4.38; 6.33)	5.30 (4.52; 6.20)	*NS*	*NS*
High-density lipoprotein cholesterol (mmol/L)		1.48 (1.14; 1.92)	1.36 (1.07; 1.76)	0.03	*NS*
Low-density lipoprotein cholesterol (mmol/L)		3.34 (2.64; 4.23)	3.58 (2.69; 4.41)	*NS*	*NS*
Triglycerides (mmol/L)		1.22 (0.90; 1.79)	1.32 (0.92; 1.78)	*NS*	*NS*
Dietary intake (kJ)		6620 (5056; 9265)	7432 (5294; 9283)	*NS*	*NS*

Adjusted for age, waist circumference, and LDL-c. Abbreviations: BMI, body mass index; BPM, beats per minute; CRP, C-reactive protein; mmHg, millimeters of mercury; WHR, waist hip ratio. Data presented as median (25th and 75th percentiles) for continuous data and number of observations (percentage) for categorical data. BMI categories’ cut-off values: Underweight <18.5 kg/m^2^; healthy 18.5–24.9 kg/m^2^; overweight 24.9–29.9 kg/m^2^; obese >29.9 kg/m^2^. Values presented in accordance with the International System of Units: to convert kJ to Cal multiply by 0.24.

**Table 3:** Genetic predisposition to develop insufficient/deficient 25(OH)D combined with elevated CRP concentrations adjusting for age, low-density lipoprotein cholesterol (LDL-c), and waist circumference.

SNP ID	Allele	Control Phenotypes	%	Case Phenotypes	%	Odds Ratio (95% CI)	*p*-Value
rs2794520	C/C	166	57.4	141	66.8		0.03
	C/T–T/T	123	42.6	70	33.2	0.65 (0.44–0.95)	
rs2808630	T/T	198	68.5	154	71.6		*NS*
	C/T–C/C	91	31.5	61	28.4	0.79 (0.53–1.18)	
rs3093068	C/C	123	42.9	72	33.5		0.03
	C/G–G/G	164	57.1	143	66.5	1.54 (1.05–2.26)	
rs1205	C/C	171	59.2	142	66		*NS*
	C/T–T/T	118	40.8	73	34	0.72 (0.49–1.05)	
rs1130864	C/C	216	74.7	170	79.1		*NS*
	C/T–T/T	73	25.3	45	20.9	0.86 (0.56–1.34)	
rs1800947	C/C	289	100	213	99.1		*NS*
	C/G	0	0	2	0.9	0	
rs1417943	A/A	277	95.8	203	95.3		*NS*
	A/T	12	4.2	10	4.7	1.7 (0.70–4.13)	
rs3093062	G/G	214	74.3	137	63.7		0.02
	A/G–A/A	74	25.7	78	36.3	1.64 (1.10–2.45)	
rs3093058	A/A	215	74.4	135	63.4		0.01
	A/T–T/T	74	25.6	78	36.6	1.67 (1.12–2.50)	
rs2027471	T/T	167	57.8	142	66		0.05
	A/T–A/A	122	42.2	73	34	0.68 (0.46–1.00)	
rs1341665	G/G	167	57.8	142	66		0.05
	A/G–A/A	122	42.2	73	34	0.68 (0.46–1.00)	
rs7553007	G/G	164	56.7	141	65.6		0.04
	A/G–A/A	125	43.3	74	34.4	0.67 (0.46–0.98)	

Cases are those presenting with the phenotype of deficient or insufficient 25(OH)D together with elevated (>3 mg/L) CRP concentrations. Controls were individuals with normal CRP and/or sufficient 25(OH)D concentrations. The reference group comprised those homozygotes for the wild-type allele. Abbreviations: 25(OH)D, 25-hydroxyvitamin D; A, adenine; C, cytosine; CI, confidence interval; G, guanine; rs, reference SNP cluster ID; T, thymine.

## Supplementary Materials

The following are available online at www.mdpi.com/1660-4601/15/1/111/s1. Table S1. Environment-SNP associations with CRP concentrations based on different genetic models of inheritance. Table S2. Demographic markers associated with differing 25(OH)D status.
