# Supplementary material for: CRP Genotypes Predict Increased Risk to Co-Present with Low Vitamin D and Elevated CRP in a Group of Healthy Black South African Women
Source: Int J Environ Res Public Health. 2018 Jan 10;15(1):111. doi: 10.3390/ijerph15010111 (PMC5800210; doi:10.3390/ijerph15010111)
Supplement: Supplementary file 1 [file ijerph-15-00111-s001.pdf]

CRP Genotypes Predict Increased Risk to Co-Present with Low Vitamin D and Elevated CRP in a Group of Healthy Black South African Women

Pieter H. Myburgh, G. Wayne Towers, Iolanthé M. Kruger and Cornelie Nienaber-Rousseau

Supplementary Material

Supplementary Table 1. Environment-SNP associations with CRP concentrations based on different genetic models of inheritance.

SNP ID	Allele	Genetic model	n	Mean adjusted CRP (mg/L)	Standard error	Association with CRP	*p*-value	AIC
**rs2794520**	C/C	Co-dominant	307	4.48	1.08	Lowered CRP	0.03	1665
	C/T		162	3.25	1.12			
	T/T		31	2.94	1.25			
	C/C	Dominant	307	4.48	1.08		0.01	1663
	C/T–T/T		193	3.2	1.11			
**rs2808630**	T/T	Dominant	352	4.02	1.07		0.62	
	C/T–C/C		152	3.82	1.13			
**rs3093068**	C/C	Co-dominant	195	3.34	1.11	Increased CRP	0.04	1670
	C/G		243	4.31	1.09			
	G/G		64	5.04	1.19			
	C/C	Dominant	195	3.34	1.11		0.02	1668
	C/G–G/G		307	4.45	1.08			
**rs1205**	C/C	Dominant	313	4.43	1.08	Lowered CRP	0.02	1676
	C/T–T/T		191	3.22	1.11			
	C/C–T/T	Over-dominant	343	4.26	1.08		0.04	1677
	C/T		161	3.38	1.12			
**rs1130864**	C/C	Dominant	386	4.22	1.07		0.25	
	C/T–T/T		118	3.21	1.13			
**rs1800947**	C/C	Co-dominant	502	3.95	1.06		0.68	
	C/G		2	8.03	1.62			
**rs1417938**	A/A	Co-dominant	480	4.01	1.07		0.94	
	A/T		22	2.97	1.4			
**rs3093062**	G/G	Dominant	351	3.43	1.08	Increased CRP	0	
	A/G–A/A		152	5.64	1.11			
**rs3093058**	A/A	Dominant	350	3.38	1.08	Increased CRP	0	
	A/T–T/T		152	5.64	1.11			
**rs2027471**	T/T	Dominant	309	4.49	1.08	Lowered CRP	0.01	
	A/T–A/A		195	3.24	1.11			
**rs1341665**	G/G	Dominant	309	4.49	1.08	Lowered CRP	0.01	
	A/G–A/A		195	3.24	1.11			
**rs7553007**	G/G	Dominant	305	4.53	1.08	Lowered CRP	0.01	
	A/G–A/A		199	3.22	1.11			

Abbreviation: A, adenine; AIC, Akaike Information Criterion; CRP, C-reactive protein; C, cytosine; G, guanine; SNP, single nucleotide polymorphism; rs, reference SNP cluster ID; T, thymine.

Although rural (Location B) and urban (Location A) participants had similar distributions stratified to 25(OH)D status, rural dwellers had higher median concentrations of 25(OH)D (69.4 nmol/L compared to 66.6 nmol/L, $p < 0.05$) and lower median CRP concentrations (3.58 mg/L vs 4.78 mg/L, p < 0.05) compared to their urban counterparts. Rural women were, however, significantly younger (median age 53 compared to 56 years), with significantly lower waist circumferences (81 cm compared to 83.3 cm). Correcting for the effects of age on 25(OH)D concentration, and waist circumference on lnCRP concentration, resulted in the rural–urban differences falling away. All HIV-positive individuals (n = 41) were first diagnosed during this study, and both 25(OH)D and CRP concentrations were similar for the HIV-positive and -negative women (whole group medians excluding HIV-positive individuals 25(OH)D: 68.2 nmol/L; CRP: 4.27 mg/L; $p > 0.05$ observed for both 25(OH)D and CRP concentrations when comparing the whole cohort and the cohort excluding HIV-positive individuals). HIV-positive subjects were, therefore, not excluded from further analyses. Smokers had significantly lower 25(OH)D concentrations. However, smoking was found not to be a significant predictor of 25(OH)D concentrations when modeled via linear regression analysis. Vitamin D intake from nutritional sources was low across all three categories of 25(OH)D status, with none of the participants ingesting the recommended 15 μg/day.

Supplementary Table 2. Demographic markers associated with differing 25(OH)D status.

Variable	Deficient <50 nmol/L (n = 81; 16.0%)	Insufficient 50—75 nmol/L (n = 250; 49.5%)	Sufficient >75 nmol/L (n = 174; 34.5%)	*p*-Value
Urban/Rural	51 (63.0%)/30 (37.0%)	132 (52.8%)/118 (47.2%)	87 (50.0%)/87 (50.0%)	*NS*
Age	57.0 [50.0;61.0][a]	55.0 [50.0;62.0][a]	52.5 [49.0;58.0][b]	0.001
Smoking status: Former/Current/Abstainer	2 (2.47%)/53 (65.4%)/26 (32.1%)	8 (3.21%)/103 (41.4%)/138 (55.4%)	3 (1.74%)/78 (45.3%)/91 (52.9%)	0.003
HIV-positive/negative	5 (6.25%)/75 (93.8%)	19 (7.63%)/230 (92.4%)	17 (9.77%)/157 (90.2%)	*NS*
Vitamin D intake (μg/day)	2.12 [1.17;3.49]	1.98 [0.98;3.57]	2.03 [0.99;3.35]	*NS*

Data presented as median [25th and 75th percentiles] for continuous data and number of observations (percentage) for categorical data. Abbreviations: 25(OH)D, 25-hydroxyvitamin D; HIV, human immune deficiency virus; NS, not significant ($p > 0.05$).
